# Annotations

**Published:** 1889-03-02

**Authors:** 


### Annotations.

**Modern Neros and their Fiddles.**

Ex nihilo nihil fit: Out of nothing nothing can come. The work of a man or of a nation is strictly proportioned to the brain power and physical energy of the man or the nation. It is entirely true that "Providence is always on the side of the strong battalions." Not necessarily on the side of the most numerous, but certainly on that of those who have most of the particular kind of strength needed at the moment, whether it be moral, intellectual, or physical. Deterioration is the way to destruction. Than this nothing is more certain. Physical deterioration is the prelude and accompaniment of mental and moral deterioration. This is as true as the verities of arithmetic. We build ships and cast guns for national defence. We do that because we see certain gross dangers which not to see would prove ourselves to be blinder than the moles. But with all our science we do not seem to be able to see far more destructive because far more insidious dangers close at hand. Nero is credited with having fiddled whilst Rome was burning, and he is held up to the contempt of schoolboys as a wretched imbecile on that account. Many a man, nay, whole cities and countries of men, not only fiddle, but do every other kind of foolish and trifling thing, whilst their personal and general habits are destroying their physique, and with it their brains and morals. Public opinion needs to be awakened and instructed on this question. Every physiologist who sees what is and knows what might and ought to be the condition of the health and energy of the people should speak in the tones of a prophet. Under the present conditions of modern life, normal health in large towns is impossible. From the age of thirty-five to sixty the average middle-class man is in no better case than a slave or a beast of burden, and is being slowly but surely ground to death exactly as they are. We want some health resources for the middle-aged; for those Atlases who bear the tremendous weight of the modern world upon their shoulders. Drugs are but feeble aids; summer holidays, hydropathic establishments, massage manipulations—these and their like are but palliatives and often miserable subterfuges. The axe must be laid to the root of the tree. New ways of life are imperatively demanded. The country schoolboy knows no illness, because all his muscles are developed by every variety of exertion, because his blood is purified by pure air and sunlight, because his brain is kept active, joyous, and wholesome by moderate work and abundant play, because his internal organs and his secreting glands are kept in activity and efficiency by his pure blood and his manifold and various exercises, because appetite, good digestion, and health are the fruits of his way of life. All these conditions, modified to suit middle-age are indispensable to robust health. If we were really the resolute and intelligent people we think ourselves, we should never be content until we had secured them in abundance for every district of every large town. Mr. Gladstone is a living object lesson of the value of carrying the physical habits and joyous carelessness of boyhood into middle life. Where is the British Bismarck who shall give unity, energy, and stability to the national health?

**Child Suicides.**

All newspaper readers have seen the accounts of the two poor girls of sixteen or seventeen who tied themselves together and drowned themselves in the canal last week. Another case, of a still more youthful would-be suicide was reported a few days ago. Benjamin Troke, a draper's assistant or apprentice, jumped into the Grand Surrey Canal, and was with difficulty saved from drowning. It appears he had attempted to defraud his employer of one penny. The two girls had been guilty of still more venial faults, and it was to avoid the consequences of these that they agreed to die together. It is plain to a medical man that these mere children had very active imaginations, conjoined with cowardly tempers. They could and did magnify alike the fault and the probable punishment, and that to such an extent that they preferred death by drowning to facing the consequences of their acts. Where is the fault? Is it the over-driving which takes place at most schools in these times; or is it in the keen competition for employment and the severe struggle with poverty which makes parents harsh and unjust to those who run the risk of losing their situations? Or are both of these conditions working towards the same end? In any case, it is plain that the stolidity and pluck and power of endurance which constitute so striking and indispensable a feature of English character are being sapped and ruined. All the education in the world will not compensate us for the loss of our grand physique and our sturdy boldness of character. All the excellence of work which competition brings with it will be of small avail if it results in the deterioration and unmanning of the workman. Somebody must look abroad with a scientific and statesman-like eye, and see where all this struggling and over brain pressure are tending. The most dangerous enemies that England has at the present time are those internal circumstances and conditions which tend to the undermining of the physique of the people, and with it, to the enfeebling of their brains and the destruction of their sturdy manhood.

**Baby-farming Superseded.**

Beatrice Stevenson, the infant daughter of a labourer at Attercliffe, has been poisoned, and nobody seems to be responsible for her death. It is desirable that everybody should clearly understand the position; for if the responsibility is not to be fixed anywhere, it is clear that a new method of getting rid of superfluous children has been accidentally discovered, and there appears to be no reason why it should not be extensively made use of. Beatrice was a fine, healthy baby; but having been overtaken by some childish ailment, she was fretful and troublesome. The father, John Stevenson, does not appear to have thought of calling in a medical man, but to have sent to the chemist for something to soothe the child. So far as we know the chemist never saw the baby, and probably had not the faintest idea what might be the matter with her. He did know, however, that he had on his shelf one all-powerful sedative which would "quiet" her, whatever might be the nature of her ailment. He did not perhaps know how deadly the soothing opium is to most young children, and what a frightful risk he was running in supplying the potent poison to ignorant persons for their use without restraint. So far as the report shows he knew nothing at all about the case, except that the parents wanted the child "quieted," and that he knew how to "quiet" it. He sent the "soothing syrup," probably without a poison label on the bottle, and the result was similar to what has taken place on hundreds of like occasions. The child took the medicine, and was speedily "quieted" for ever. No mention is made of any punishment to be meted out to anybody. The father escapes scot free, though why he should is not evident, for if he had not money to pay a medical man he could have called in the parish doctor. The chemist was mildly told that he should have made sure beforehand that those in charge of the child could read, and so have prevented an overdose. He, therefore, escapes also without even a reprimand. Manifestly infant life is cheap at Attercliffe. It is possible, however, that it may become too cheap. There are plenty of people in the neighbourhood of Sheffield and Rotherham who find their children burdensome: here is a very easy way of making their families smaller. Chemists have always been very accommodating, and if coroners are now going to be equally civil there seems no reason why the infant population should not be cleared off in hundreds by such an easy and safe method of "misadventure." Baby-farming is coarse and revolting in comparison with the new process, and has the disadvantage of being slow. "Misadventure" may kill its thousands, and painlessly too, whilst baby-farming is busy with its units. Who says that ours is not an age of progress?

